# Active disturbance rejection-based decentralised sensor fault-tolerant control in DC microgrids

**DOI:** 10.1038/s41598-026-47847-2

**Published:** 2026-04-15

**Authors:** Ahmed M. I. Mohamad, Amr M. Ibrahim, Ehab H. E. Bayoumi

**Affiliations:** 1 Department of Electrical Power & Machines, Faculty of Engineering, Ain Shams University, Cairo, 11517 Egypt; 2 Energy and Renewable Energy Engineering Department, Faculty of Engineering and Technology, The Egyptian Chinese University, Cairo, Egypt; 3 Mechatronics and Robotics Section, Department of Mechanical Engineering, Faculty of Engineering, The British University in Egypt (BUE), El Sherouk, Cairo 11837 Egypt

**Keywords:** Sensor failure, Fault-tolerant control, Active disturbance rejection control, DC microgrids, Energy science and technology, Engineering, Mathematics and computing

## Abstract

DC microgrids have become a viable solution for modern power distribution systems because they offer better control, improved efficiency, and simpler integration with renewable energy sources and energy storage systems. However, the performance of low-voltage DC microgrids can suffer from stability issues related to unpredictable sensor faults, parameter uncertainty, and equipment failure. In recent years, disturbance-rejection methods and robust control methods have been effective in improving microgrid resilience during these situations. This paper proposes a decentralized sensor fault-tolerant control approach for an islanded low-voltage DC (LVDC) microgrid using the active disturbance rejection control (ADRC). The ADRC control preserves the DC grid stability in the presence of unknown and time variant sensor faults by estimating and compensating for lumped disturbances through an extended state observer without the need for fault detection or reconfiguration of the system. A thorough mathematical model and an analytical control formulation are provided and thoroughly examined through single, consecutive, and simultaneous sensor-fault scenarios. Time-domain nonlinear simulation studies on a multi-DG DC microgrid show that the proposed controller provides better voltage regulation, faster transient recovery, and better robustness compared to other proposed methods in the literature, such as the conventional autotune PI controllers and the attractive ellipsoidal–based methods. The simulation studies’ results verified that the proposed ADRC scheme noticeably increases the reliability and resilience of the DC microgrid under realistic simulation conditions of sensor faults.

## Introduction

Low-voltage direct current (LVDC) microgrids have emerged as a smart option in power systems due to their high efficiency, inherent compatibility with renewable energy sources, and ease of integration with distributed generation and energy storage systems. They have become a key-player component of modern power systems infrastructure for applications including renewable energy integration, industrial power systems, and electrification of remote communities. This is because DC microgrids have significant advantages over their AC counterparts, for example, the absence of the reactive power term, simple control, and compatibility with modern electronic-based loads and storage systems^1^. Despite being reliable systems, the reliability of the operations of LVDC microgrids is dependent on reliable sensing and control. Measurement devices such as voltage/current sensors are subject to degradation with time, in the form of bias, drift, or loss of signal, which can lead to deterioration in the system performance, inadequate regulation, and potential overall system failure^2–4^. As a result, ensuring resilience in the operation of systems considering sensor faults has recently become a significant area of importance in DC microgrid control. Proportional–Integral (PI) controllers are commonly used for regulating the DC grid’s bus voltage because of their simplicity and ease of design and implementation^5^. However, PI controllers in practical systems can be very fragile components and can often fail due to parameter variation, non-linearity, or due to sensor measurement faults. As a result, fault-tolerant control (FTC) solutions are becoming a rapidly growing area of interest.

These methods are aimed at keeping the system stable despite faulty or missing sensor data^6–8^. There are generally two classes of fault-tolerant control (FTC) strategies for DC microgrids based on the type of FTC employed: passive and active FTC methods. Passive FTC methods utilize fixed and robust controllers without the need to detect faults to preserve the system stability once a fault has occurred. Therefore, in passive FTC schemes, faults are modeled as bounded uncertainty or bounded disturbances and robust control methods are implemented to mitigate their effects on the system performance. Passive FTC approaches have certain advantages, such as robustness, simplicity, and low operational cost. However, these methods seem a bit conservative because they operate close to normal conditions they cannot adapt well to severe faults. The concept of the active methods is usually based on presenting explicit methods for fault detection, diagnosis, and compensation mechanisms to maintain control performance during sensor faults. Active FTC strategies rely on observer-based methods like sliding mode observers (SMO) to reconstruct the state of the system and provide fault signal estimates in real time; these approaches can often provide better performance because they can deal with more complex faults compared to passive FTC^9,10^.

It must be pointed out, however, that passive fault-tolerant control offers some benefits overactive methods of control in terms of design and implementation, as it does not rely on fault detection, fault isolation, or reconfiguration of the controller. In addition, it does not require many computational resources, which is important when the processing capabilities are limited. Moreover, passive FTC usually provides a faster response under faulty conditions because the controller is initially designed to be robust and does not have to wait for a specific decision based on the fault diagnosis process. The FTC schemes can also be classified according to their architecture, either centralized or decentralized and distributed. Centralized FTC architectures utilize a supervisory controller that gathers observations of the system state and computes control actions for all agents, enabling control coordination, which can create a single point of failure and requires careful attention to communication links. Decentralized architectures rely on local measurements and, therefore, provide greater robustness in the case of communication failures. The distributed architectures rely on algorithms to enable neighboring agents to work together to achieve overall stability of the system, while minimizing any necessary communication with a central controller. However, that may also serve as a single point of failure to the system. Although these highly resilient architectures afford greater flexibility in environments with communication disruptions, distributed architectures involve higher computing and communication costs because they require agents to exchange information and also may be subject to cyber threats and false data injection attacks^11–17^.

Quantitative evaluations reported in the literature indicate that passive FTC strategies typically maintain stability within bounded disturbance ranges but may exhibit settling times exceeding 1–2 s under severe faults^9,15^. Active observer-based strategies improve disturbance rejection but often require fault detection delays ranging from several milliseconds to hundreds of milliseconds, depending on algorithm complexity^6,14^. Furthermore, centralized architectures introduce communication latency and potential single-point failure risks, whereas decentralized approaches eliminate communication dependency at the expense of requiring robust local disturbance handling. These quantitative observations highlight the need for a decentralized framework capable of fast disturbance estimation without fault detection delay, which motivates the ADRC-based structure proposed in this work.

More recently, the active disturbance rejection control (ADRC) has developed as a promising alternative to the traditional approaches. ADRC captures the simplicity of error-driven control like PID with the power of modern state observers to actively estimate and reject both internal and external disturbances. In this control method, nonlinear feedback is utilized to manage model uncertainties and becomes an effective and useful digital control approach with significant advantages over traditional methods. The Extended State Observer (ESO) in ADRC reconstructs both system states and the total disturbances affecting the states, such as the disturbance resulting from faults in the sensors, allowing the controller to preserve the system stability and provide acceptable dynamics performance^18,19^.

Due to its simplicity, robustness, and fast dynamic response, the ADRC has been widely used in different power system applications, as reported in the literature. In^20^, the authors presented a control strategy for mitigating voltage imbalance in microgrids using the attracting ellipsoid method. This was achieved by treating negative-sequence components as external disturbances, yielding a robust voltage tracking under unbalanced and faulty conditions. In^21^, the authors proposed a parameter tuning formula for a second-order linear active disturbance rejection controller based on the step response curves of the system under control.

In the DC microgrids field of study, the authors in^22^ proposed a linear active disturbance rejection control method for buck and boost DC/DC converters in DC microgrids operating as grid-forming units. The controller integrates an augmented Kalman filter, adaptive reference trajectory generator, and linear quadratic regulator feedback to handle different system disturbances. In^23^, a fractional-order linear active disturbance rejection control scheme has been proposed to overcome the slow response and low accuracy of conventional ADRC in buck DC/DC converters. In^24^, an actor-critic-based linear active disturbance rejection control strategy was proposed for DC-DC converters in microgrids to enhance system stability and anti-disturbance capability. This was achieved via self-tuning of ADRC parameters, allowing for adaptive control under different dynamic conditions.

In^25^, an ADRC-based control strategy is proposed for DC microgrids to ensure bus voltage restoration and accurate power sharing under communication disturbances and delays. The method achieves voltage–power decoupling and minimizes communication requirements among units. In^26^, the authors proposed an ADRC-based control strategy for the bidirectional DC-DC converter in a modular multilevel converter-based battery energy storage system within AC/DC hybrid networks to enhance the voltage and current stability under load variations and grid unbalances.

Based on the above literature survey, PI controllers remain the most widely adopted solution for DC bus voltage regulation due to their simplicity and ease of implementation. However, their effectiveness deteriorates when the system is subjected to parameter variations, nonlinearities, or measurement faults. Although various fault-tolerant control (FTC) strategies have been proposed for LVDC microgrids, however, ADRC has never been implemented in DC grids for FTC, to the authors’ knowledge, leaving an opportunity for developing a more robust and disturbance-driven method. Therefore, this paper proposes an ADRC-based sensor fault-tolerant control framework for LVDC microgrids. The main contributions are summarized as follows:

1. Development of an ADRC-based fault-tolerant control framework capable of estimating and compensating for both external disturbances and sensor faults in real time.
2. Presenting a complete mathematical model along with the full analytical control equations for an islanded low-voltage DC microgrid.
3. Presenting a comprehensive comparative study for the proposed ADRC method, autotune PI, and (Attracting Ellipsoid Method) AEM controllers under various operating conditions, to assess the dynamic response, robustness, and computational complexity of each method.

Although several FTC strategies have been reported for DC microgrids, most of them either rely on accurate plant modelling, explicit fault detection mechanisms, or controller reconfiguration once a fault is identified. These requirements increase implementation complexity and may introduce delays during the fault diagnosis phase. In contrast, the proposed ADRC-based structure does not treat sensor faults as events that must be detected and isolated. Instead, it models them as part of the lumped disturbance estimated online through the extended state observer. This removes the structural dependency on fault diagnosis and avoids controller switching or gain reconfiguration. Therefore, the contribution of this work is not merely applying ADRC to DC microgrids but reformulating the sensor fault problem into a disturbance-rejection framework, which simplifies implementation while improving resilience.

The remainder of this paper is structured as follows. The problem definition and system dynamics modelling configuration are in “Problem definition and system dynamics modelling”. The active disturbance rejection control (ADRC) architecture details are given in “Active disturbance rejection control (ADRC) architecture”. Detailed offline and real-time simulations and a comprehensive discussion are presented in “Results” and “Discussion” to evaluate and assess the proposed method. Finally, the findings and conclusions are summarized in “Conclusion”.

## Problem definition and system dynamics modelling

**Fig. 1:**
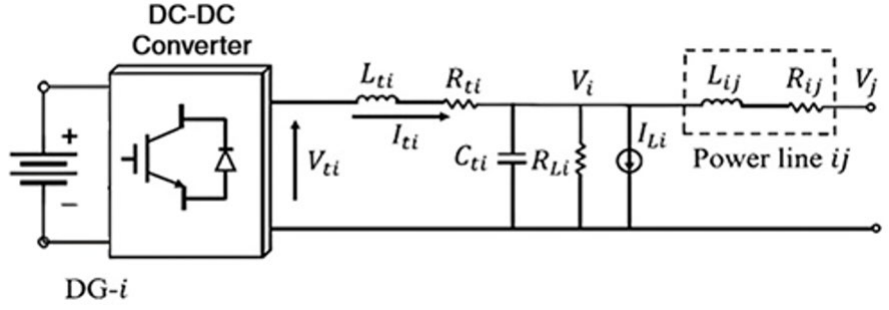
The electrical diagram for the *i*-th distributed generator.

This paper investigates a DC microgrid (MG) containing N distributed generators (DGs) interconnected via DC lines. Figure 1 shows the block diagram of the *i*th DG connected to a DC microgrid.

As shown in Fig. 1, each DG unit consists of a DC power supply block (representing the PV source integrated with battery storage), followed by a DC–DC converter stage. The DG unit is modeled as a DC voltage source, DC/DC converter, *R-L-C* filter, and resistive load (*R*_*Li*_). The renewable energy sources have an inherently intermittent nature; however, this is usually overcome by adding energy storage systems (ESS) to ensure reliable operation and power supply. For these reasons, an assumption in the study is to model the PV sources with a battery storage system, which will allow for the DGs to demonstrate constant output voltage and can be analyzed in steady-state operating mode.

Applying Kirchhoff’s voltage and current laws (KVL/KCL) to DG − *i* yields the following dynamic model:

1 $$\:\left\{\begin{array}{c}\frac{d{V}_{i}}{dt}=\frac{1}{{C}_{ti}}\:\:{I}_{ti}-\frac{1}{{C}_{ti}}\:\:{I}_{Li}+\frac{1}{{C}_{ti}}\:{I}_{ij}\\\:\frac{d{I}_{ti}}{dt}=-\frac{1}{{L}_{ti}}\:\:{V}_{i}-\frac{{R}_{ti}}{{L}_{ti}}\:\:{I}_{ti}+\frac{1}{{L}_{ti}}\:\:{V}_{ti}\\\:Line\:\:ij:\:\:\frac{d{I}_{ij}}{dt}=-\frac{{R}_{ij}}{{L}_{ij}}\:\:{I}_{ij}+\frac{1}{{L}_{ij}}\:\:{V}_{j}-\frac{1}{{L}_{ij}}\:\:{V}_{i}\end{array}\right.$$

The notation used to define the *i*th DG dynamics is as follows: *V*_*i*_​ and *I*_*ti*​_ represent the *i*th DG capacitor voltage and output current, respectively. At the same time, *V*_*ti*​_ is the converter control signal. The constant parameters *R*_*ti​*_, *L*_*ti*​_, and *C*_*ti*_​ characterize the properties of the filter. Furthermore, *R*_*ij*_​ and *L*_*ij​*_ denote the power line impedance connecting DG-i to DG − *j*, and *V*_*j*_ is the capacitor voltage of DG-j. The DC feeders connecting the DGs are modelled with quasi-stationary dynamics (i.e., *dI*_*ij*_*​/dt* = 0) in Eq. (1)^15^. This simplification is justified because the line inductance *L*_*ij*_​ in DC systems is typically negligible, allowing the associated line dynamics to be safely disregarded.

2 $$\:\left\{\begin{array}{c}\therefore\:\:\:{I}_{ij}=\frac{{V}_{j}-{V}_{i}}{{R}_{ij}}\\\:\frac{d{I}_{ti}}{dt}=-\frac{1\:}{{L}_{ti\:}}{V}_{i}-\frac{{R}_{ti}}{{L}_{ti}}\:{I}_{ti}+\frac{1}{{L}_{ti\:}}\:{V}_{ti}\\\:\frac{d{V}_{i}}{dt}=\frac{1}{{C}_{ti}}{I}_{ti}-\frac{1}{{C}_{ti}}\:\:{I}_{Li}+\frac{1}{{C}_{ti}{R}_{ij}}{V}_{j}-\frac{1}{{C}_{ti\:}{{R}_{i}}_{j}}\:\:{V}_{i}\:\end{array}\right.$$

For *N* DGs within the islanded DC MG (Fig. 1), all can be represented by the following equivalent state-space equations:

3 $$\:{\dot{x}}_{i}={A}_{ii}{x}_{i}+{B}_{i}\:{u}_{i}+{D}_{i}\:{w}_{i},\:\:{y}_{i}={C}_{i}{x}_{i}$$

The state-space representation utilizes a vector $\:x={\left[{V}_{i}\:\:{I}_{ti\:}\right]}^{{\prime\:}}$ for the system states and $\:{u}_{i}={V}_{ti}$ for the control inputs. The measurable output is given by *y*_*i*_, which is taken to be equal to the corresponding state vector, xi​. The matrices governing these dynamics, *A*_*ij*_​ and *B*_*i*_​, are defined below:

4 $$\:\begin{array}{c}{A}_{ii}=\left[\begin{array}{cc}-\frac{1}{{C}_{i}}{\sum\:}_{j}\frac{1}{{R}_{ij}}-\frac{1}{{R}_{i}{C}_{ti}}&\:\frac{1}{{C}_{ti}}\\\:-\frac{1}{{L}_{ti}}&\:-\frac{{R}_{ti}}{{L}_{ti}}\end{array}\right]\\\:{A}_{ij}=\left[\begin{array}{cc}\frac{1}{{R}_{ij}{C}_{ti}}&\:0\\\:0&\:0\end{array}\right]\\\:{B}_{i}=\left[\begin{array}{c}0\\\:\frac{1}{{L}_{ti}}\end{array}\right]\\\:{C}_{i}=\left[\begin{array}{cc}1&\:0\\\:0&\:1\end{array}\right]\end{array}$$

The external disturbance is:

5 $$\:{D}_{i}=[{A}_{i1}..{O}_{ii}..{A}_{iN}],$$

**Fig. 2:**
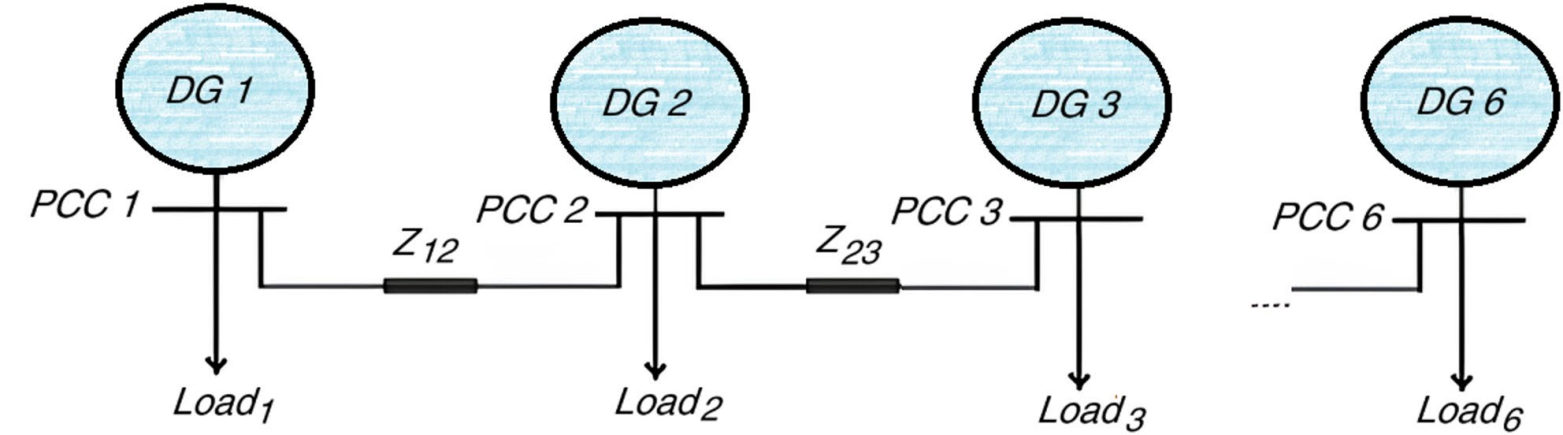
A six-DG islanded DC microgrid.

To align with prevalent distribution network architectures, the studied system is radially configured. The complete islanded DC microgrid of the studied system, consisting of *N* = 6 DGs, is presented in Fig. 2.

The electrical parameters for the distributed generation units and the distribution lines are summarized in Tables 1 and 2.

**Table 1:** Electrical Parameters of the Microgrid^15^.

DGs	Parameters of the DC-DC converter			Shunt capacitance C_t_ (mF)	Load *R*(Ω)	Power rating (W)
	*R*_t_ (Ω)		L_t_ (mH)			
DG_1_	7.22		72.2	25	160	1200
DG_2_	7.22		72.2	32	80	600
DG_3_	7.22		72.2	25	120	900
DG_4_	7.22		72.2	30	160	1200
DG_5_	7.22		72.2	18	100	800
DG_6_	7.22		72.2	12	120	900
	*V*_*dc*_ (DC bus voltage) = 100 V			*f*_*sw*_ (Switching frequency) = 40 kHz		

Noted that the RLC parameters presented in this table correspond to aggregated filter dynamics used to represent practical LVDC microgrid converters rather than standalone low-power laboratory converters. Although the rated DG power is 1200 W at 100 V, the relatively higher inductance and capacitance values are selected to ensure adequate voltage ripple suppression, current smoothing, and dynamic stability in interconnected multi-DG operation. These parameter magnitudes are consistent with previously reported DC microgrid benchmark systems^15^, ensuring comparability of results.

**Table 2:** Parameters of distribution lines^15^.

Line impedance (Z_ij_)	Line resistance (*R*_ij_)			Line inductance (L_ij_)		
	*r*_ij_ (Ω/m)	Cable length (m)	*R*_ij_ (Ω)	l_ij_ (µH/m)	Cable length (m)	L_ij_ (µH)
$\:{Z}_{12}$	0.05	180	9	1.8	180	324
$\:{Z}_{23}$	0.05	240	12	1.8	240	432
$\:{Z}_{34}$	0.05	300	15	1.8	300	540
$\:{Z}_{45}$	0.05	240	12	1.8	240	432
$\:{Z}_{56}$	0.05	264	13.2	1.8	264	475.2

Although the case study considers a six-DG radial microgrid for clarity and benchmarking consistency with previous literature, the state-space in Eq. (3) is expressed as a general N-DG system. Since each ADRC controller is designed locally for each DG unit using only local voltage and current measurements, no centralized coordination or global system model is required. Therefore, expanding the system to a larger number of DGs does not alter the controller structure; it only increases the dimensionality of the interconnection matrix Aij. This property ensures structural scalability and applicability to arbitrary DC microgrid topologies, including radial, meshed, or hybrid configurations.

## Active disturbance rejection control (ADRC) architecture

### Principle of ADRC

The Active Disturbance Rejection Control (ADRC) is a non-model-based control strategy developed to facilitate system resilience with real-time estimation and compensation for the lumped disturbance (including internal dynamics and external disturbances). The ADRC is considered a good candidate to deal with even complex and uncertain situations. The control action uses three specific modules, the Tracking Differentiator (TD), Extended State Observer (ESO), and Nonlinear State Error Feedback (NLSEF), in which all modules work together to achieve precise control and an effective performance.

### ADRC structure

#### Tracking differentiator (TD)

The Tracking Differentiator (TD) module generates continuous reference signals and their differential estimations while filtering the higher frequency signals. Controlling the reference input’s transition rate allows the TD to enhance closed loop stability and avoid the system from being subjected to underdamped oscillations. These properties are useful in cases where the setpoint changes frequently or suddenly.

#### Extended state observer (ESO)

The ESO represents the core of the ADRC and was developed to monitor key system variables and all the disturbances in real-time. The primary features of the ESO are its ability to redefine the overall disturbance, either due to internal or external uncertainties, in terms of state variables. The employment of these state-based variables cancels out the disturbance effects dynamically. The ESO relies on a continuous adaptation process to ensure precise and robust estimation.

#### Nonlinear state error feedback (NLSEF)

The third component, known as the NLSEF, is responsible for implementing the corrective control action based on the estimated states and resulting system errors. The NLSEF can integrate nonlinear specific nonlinear functions other than linear feedback, which yields higher accuracy and adaptability. This is more beneficial, particularly when dealing with systems that are highly nonlinear or associated with time-varying dynamics.

### ADRC mathematical modelling

Although typical ADRC designs rely on employing TD for reducing noise and for obtaining smooth reference derivatives, this work proposes a simplified ADRC variant, which assumes a clean, noise-free reference input, allowing the time-domain ADRC design to be used more effectively.

For an *n*th order linear plant defined by the output y, the control input u, and the input disturbance q, which can be represented by the following model:

6 $$\:{y}^{\left(\left(n\right)\right)}\left(t\right)=q\left(t\right)+{b}_{0}u\left(t\right)$$

The total disturbance, denoted by q, incorporates unmodeled system dynamics (model uncertainties) and external perturbations. It is commonly noted that the second-order system model is given by:

7 $$\:\:P\left(s\right)=\frac{K}{{T}^{2}{s}^{2}+2DTs+1}$$

These configurations are frequently used in wide industrial applications. Consequently, the dynamics of the plant and the overall disturbance can be closely approximated by the following expression:

8 $$\:{T}^{2\:}\ddot{y}\left(t\right)+2DT\dot{y}\left(t\right)+y\left(t\right)=Ku\left(t\right)+d\left(t\right)$$

With $$\:{b}_{0}\cong\:K/{T}^{2}$$.

For implementation within the linear ADRC framework, a Luenberger observer is utilized to generate real-time estimations of the plant’s state variables alongside the aggregated disturbance *q*^20^:

9 $$\:\dot{\widehat{x}}\left(t\right)=\left(A-LC\right)\widehat{x}\left(t\right)+Bu\left(t\right)+Ly\left(t\right)$$

The matrices *A*,* B*, and *C* are:

10 $$\:A=\left(\begin{array}{ccc}0&\:1&\:0\\\:0&\:0&\:1\\\:0&\:0&\:0\end{array}\right),\:\:\:\:\:B=\left(\begin{array}{c}0\\\:{b}_{0}\\\:0\end{array}\right),\:\:\:C=\left(\begin{array}{ccc}1&\:0&\:0\end{array}\right)$$

**Fig. 3:**
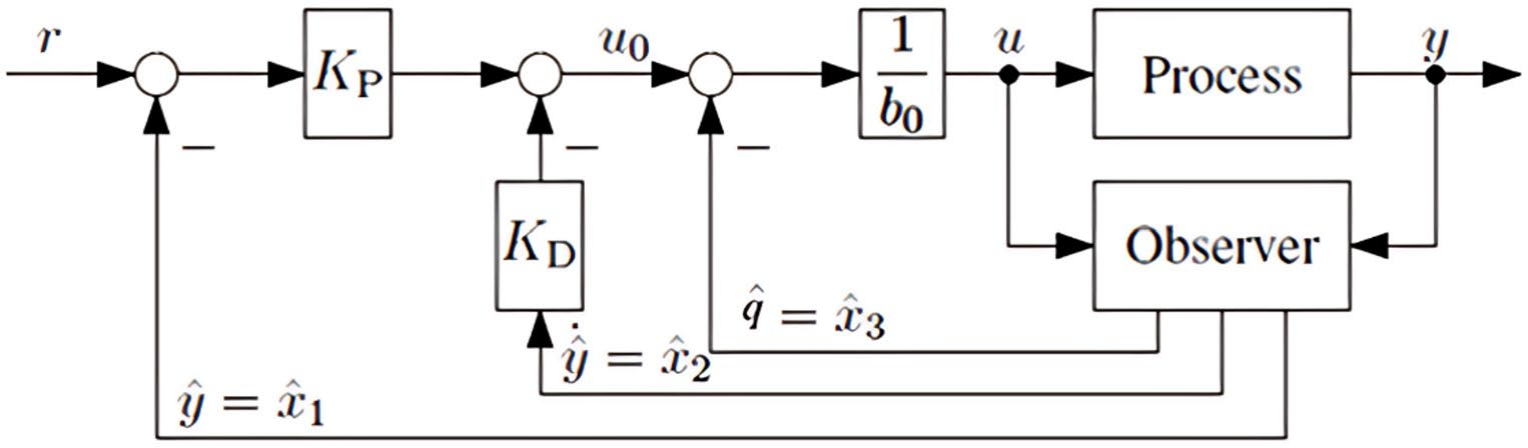
The ADRC closed-loop system.

The control input is generated by a state-space controller, which leverages the estimated state variables ($\:\widehat{x}$) to achieve disturbance rejection. The general structure of this control strategy is depicted in Fig. 3 and defined in Eq. (11) by

11 $$\:u\left(t\right)=\frac{{K}_{P}}{{b}_{0}}\:\:r\left(t\right)-{\omega\:}^{T}\widehat{x}\left(t\right)$$

Fault-tolerant control for the DC microgrid can be realized using the ADRC approach, as described by Eqs. (6)-(11). Accordingly, the feedback gain vector for the ADRC is subsequently tuned based on the dominant dynamics of the plant, as demonstrated in Eq. (12):

12 $$\:{\omega\:}^{T}=\left[\begin{array}{ccc}{K}_{P}&\:{K}_{D}&\:\frac{1}{{b}_{0}}\end{array}\right]$$

The selection of the observer gain vector L (Eq. 9) is very important, as it must ensure that the observer’s poles reside substantially to the left of the closed-loop poles within the complex s-plane. A standard practice for placement involves consolidating all closed-loop poles at a common location, s_CL_ ($\:\approx\:\frac{-4}{{T}_{s}},\:\:{T}_{s\:\:}$= desired settling time), and to position the extended state observer poles at s_ESO_ $\:\approx\:$(3 …10). s_CL_^20^.

A preliminary pole-zero system analysis confirmed a dominant second-order characteristic, which consequently validates the selection of a second-order ADRC structure for implementation. The design of this controller is straightforward, requiring only the parameter *b*_*0*_​, which can be readily obtained from the step response of the simplified second-order model representing the original DC microgrid proposed system.

By determining the system’s DC gain and dominant time constant, *b*_*0*_ = 2.037. For a chosen closed-loop settling time Ts = 0.2 s, the observer poles are placed around − 185. The observer’s gain is $\:L=\left[\:381\:,\:12456\:,\:\:7.561e+05\right]{\prime\:}$. The parameter *b*_*0*_ represents an approximation of the process dynamics. The controller parameters, *K*_*P*_ and *K*_*D*_, can be selected through pole placement techniques to achieve a specified settling time for the closed-loop system. For the ADRC implementation, the initial controller parameters were first established using a standard heuristic tuning methodology^21^. These preliminary values were then significantly refined through a metaheuristic optimization approach, specifically Particle Swarm Optimization (PSO), as documented in sources^27^. The resulting, optimized controller gains are:$\:{K}_{P}=732.24,{\:\mathrm{a}\mathrm{n}\mathrm{d}\:K}_{D}=\:86.59$. Table 3 summarizes the design process for the ADRC of the DC microgrid proposed system.

In the decentralized structure, each DG controller operates using its local state measurements. Interactions between DGs appear as coupling terms in the disturbance component of Eq. (8). Since ADRC estimates the total lumped disturbance, including coupling effects, through ESO, these interactions are compensated in real time.

Stability is ensured through pole-placement design. The closed-loop poles are selected to satisfy a desired settling time, while the observer poles are placed significantly further to the left in the complex plane to guarantee fast disturbance estimation. Provided that the observer bandwidth exceeds the plant bandwidth by a sufficient margin, the estimation error remains bounded, and the closed-loop system behaves as a stable second-order system. Therefore, mutual interference among DG units is treated as a bounded interconnection disturbance rather than destabilizing feedback, preserving decentralized stability.

**Table 3:** Summary of control gains and bandwidth for fast ADRC implementation.

Parameter	Symbol / value	Description	Derivation / purpose
Approximation parameter	*b*_*0*_​=2.1637	Approximation of the process dynamics	Derived from the system’s DC gain and dominant time constant
Desired settling time	*T*_*s*_​=0.2 s	Target time for the closed-loop system response	Specified performance requirement
Observer pole location	≈−60 to -200	Location of the ESO poles in the s-plane	Set significantly far left of the closed-loop poles to ensure fast observation
Observer gain vector	L =$\:\left[\:381\:,\:\:12456\:,\:\:7.561e+05\right]{\prime\:}$	Gain vector for the Luenberger observer (ESO)	Calculated based on the chosen observer pole location
Proportional gain rule	*K*_*P*_​=ω_c_^2​^ (initial value)	Proportional gain rule (initial value)	Metaheuristic tuning methodology from^27^
Derivative gain rule	*K*_*D*_=2ω_c_​ (initial value)	Derivative gain rule (initial value)	Metaheuristic tuning methodology from^27^

Note that: ω_c_ is controller bandwidth (or closed-loop bandwidth).

## Results

Figure 2 illustrates the system model under investigation that has been developed with the help of MATLAB/Simscape Electrical Toolbox. The investigated system ensured compliance with the IEEE Std 1159–2009 standard^28^, and showed strong stability, dynamic response, and steady-state performance. To assess the effectiveness of the controllers developed, simulations were conducted where sensors experienced random sensor faults in the manner of a degraded efficiency of the sensor signal (expressed as a percentage). Each of the proposed control strategies were evaluated through four unique study scenarios, permitting random additions of a level of sensor failure within a given DG unit. The controller approach was previously discussed in the section that yielded six ADRC controllers, where the controller gains are given in Table 4.

The simulation was conducted using MATLAB/Simulink with a fixed-step discrete solver to emulate real-time execution conditions. The sampling time was selected as 50 µs, which is compatible with the converter switching frequency of 40 kHz. The computational time per control cycle remained well below the sampling interval, confirming feasibility for real-time DSP or hardware implementation. Since the ADRC structure consists primarily of linear observer equations and algebraic feedback computation, its computational burden is comparable to that of a conventional controller augmented with state estimation.

**Table 4:** The proposed ADRC gains for the six DGs.

ADRC-gains	DG_1_	DG_2_	DG_3_	DG_4_	DG_5_	DG_6_
*K*_*P*_	437.52	513.78	489.45	562.34	715. 95	587.74
*K*_*D*_	52.391	49.673	63.174	48.723	64.875	56.561

A benchmark comparison with the novel technique was established by designing six controllers for the DG units shown in Fig. 2 using alternative control strategies: the decentralized auto-tuned control and the attractive ellipsoid methods. The specific design gains utilized for these six auto-tuned PI controllers and the six Attractive Ellipsoid controllers are provided in Table 5^15^ contains the detailed design specifications for the decentralized attractive ellipsoid technique. A summary overview of the methodologies for the auto-tuned control schemes is given in Remark 1.

### Remark 1

Summary of the proposed and auto-tuned control algorithms.

The proposed algorithm:

- For a given scalar α, the system matrix equations become linear; solve them by the Matlab LMI toolbox.
- Calculates the objective function *{*max _*j* =1,2_ *tr{*(*diag ϕ*_*i*,*j*_(*t*) *C*_*i*_) *Pi* (*diag ϕ*_*i*,*j*_(*t*) *C*_*i*_)*’}*,
- Updates α iteratively till the minimum of the objective function is obtained (the Matlab command fminsearch can be used).

The auto-tune PI control methodology defines controller gain settings through analysis of the system’s model or data. Implementation relies on the Simulink Control Design™ feature to automatically adjust PI gains within a Simulink platform. Operationally, the auto-tuner necessitates a linearization of the system model, subsequently calculating the PI controller gains to ensure an acceptable compromise between system robustness and required performance specifications.

To ensure objective comparison, all controllers were tuned to satisfy comparable nominal closed-loop performance specifications before fault injection. Specifically, the auto-tuned PI and Attractive Ellipsoid controllers were adjusted to achieve similar settling times and steady-state accuracy under healthy operating conditions. The ADRC gains were initially selected via heuristic rules and then optimized using PSO under identical performance indices. Therefore, the performance differences observed under fault conditions reflect structural robustness rather than unequal tuning effort.

**Table 5:** Auto-tuned PI and attractive ellipsoidal controller gains for the six DGs.

Type	DG_i_	Gain
	Controller	K_*p*_, K_i_
Auto-tuned PI	DG_1_	2.734, 50.651
	DG_2_	1.643, 24.372
	DG_3_	2.137, 21.934
	DG_4_	2.072,22.891
	DG_5_	2.952, 33.782
	DG_6_	2.851, 47.631

	Controller	K_*X*_, K_I_
Attractive ellipsoidal technique^15^	DG_1_	[-14.649, -45.078], 42.592
	DG_2_	[-59.741, -74.988], 155.51
	DG_3_	[-24.598, -55.922], 22.399
	DG_4_	[-18.248, -43.158], 52.527
	DG_5_	[-98.091, -114.53], 108.91
	DG_6_	[-85.036, -86.043], 145.12

We’ll test the proposed control method against auto-tuned PI and attractive ellipsoid control techniques across four scenarios. These scenarios involve applying random sensor faults to random DGs:

1. A random sensor degradation on one DG.
2. Consecutive sensor degradations on two DGs.
3. Simultaneous degradations on two DGs (within the design control range in^15^ ).
4. Simultaneous degradations on two DGs (outside the design control range in ^15^).

Sensor degradation was modeled as a multiplicative fault in the measurement channel. Specifically, the measured signal $\:{y}_{m}\left(t\right)$was defined as.

13 $$\:{y}_{m}\left(t\right)= \beta {\hspace{0.17em}}y\left(t\right)$$

where $\:y\left(t\right)$is the true signal and $\beta \:\left.\in\:[\mathrm{0,1}\right]\:$represents the sensor effectiveness coefficient. For example, 75% effectiveness corresponds to $\beta \:=0.75$. Random degradation events were introduced as step changes in β at the specified fault times.

### Scenario 1: one DG experiences sensor degradation

#### Case 1: sensor degradation in DG_1_

**Fig. 4:**
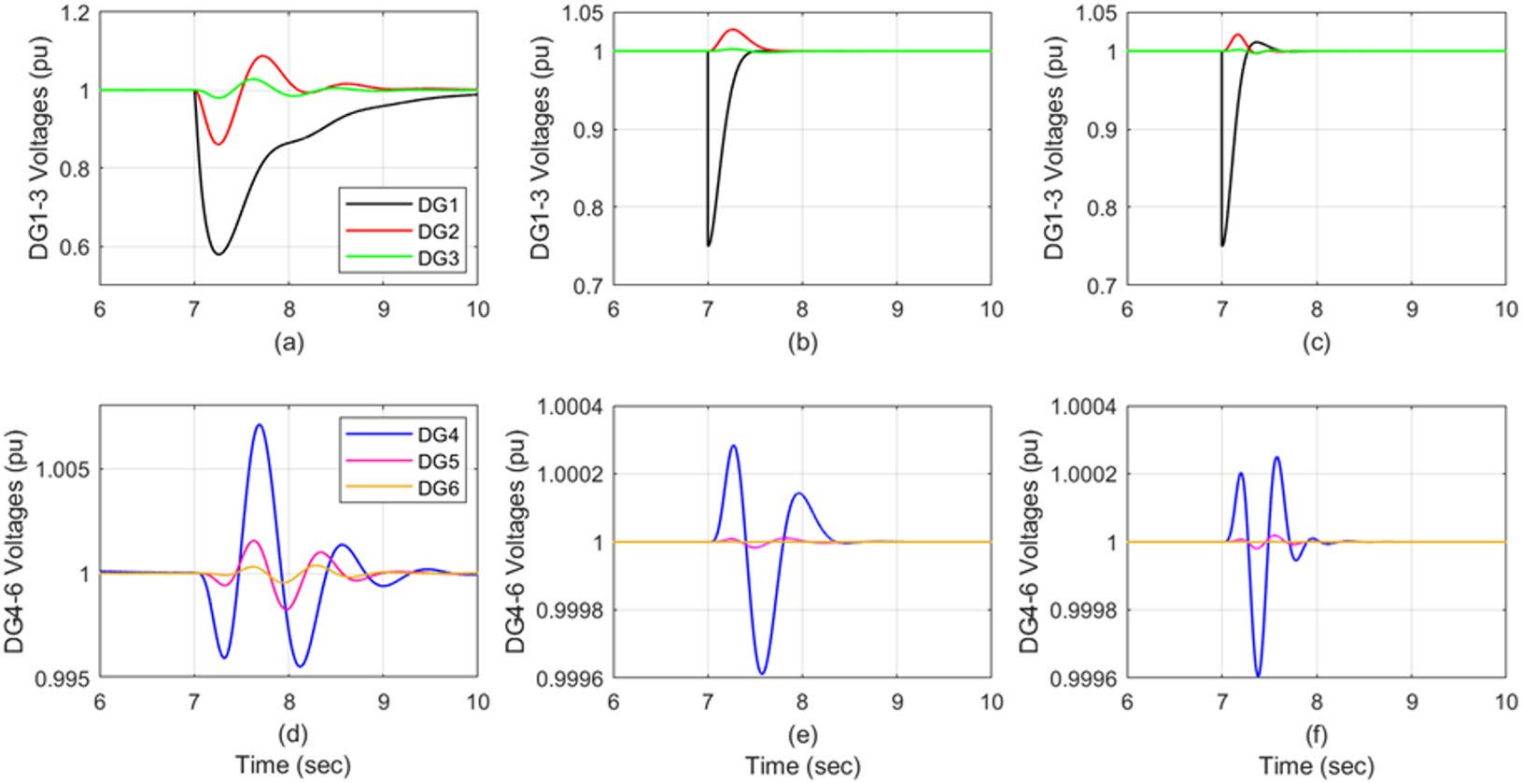
The DGs’ voltage response when DG_1_’s sensor effectiveness degrades from 100% to 75%. Auto-tune PI in (**a,d**), attractive ellipsoid in (**b,e**) and ADRC in (**c,f**).

Randomly generated sensor fault was applied to DG_1_ at t = 7s, reducing its effectiveness to 75%. Figure 4 compares the DG voltage responses following this case: Figure 4a and d show the response of the auto-tuned PI, Fig. 4b and e show the response of the attractive ellipsoid control, and Fig. 4c and f display the response of the proposed control technique’s performance.

The auto-tuned PI approach shows significantly severe impacts from the DG_1_ fault on the other five DGs. Conversely, both the attractive ellipsoid and the proposed control (ADRC) techniques effectively suppress the fault propagation, resulting in minimal, near-zero effects. Notably, the ADRC is slightly faster in its response than the attractive ellipsoid control for this scenario. Table 6 summarizes these comparative implications of DG1’s 75% sensor effectiveness across all three control techniques.

#### Case 2: sensor degradation in DG_5_

**Fig. 5:**
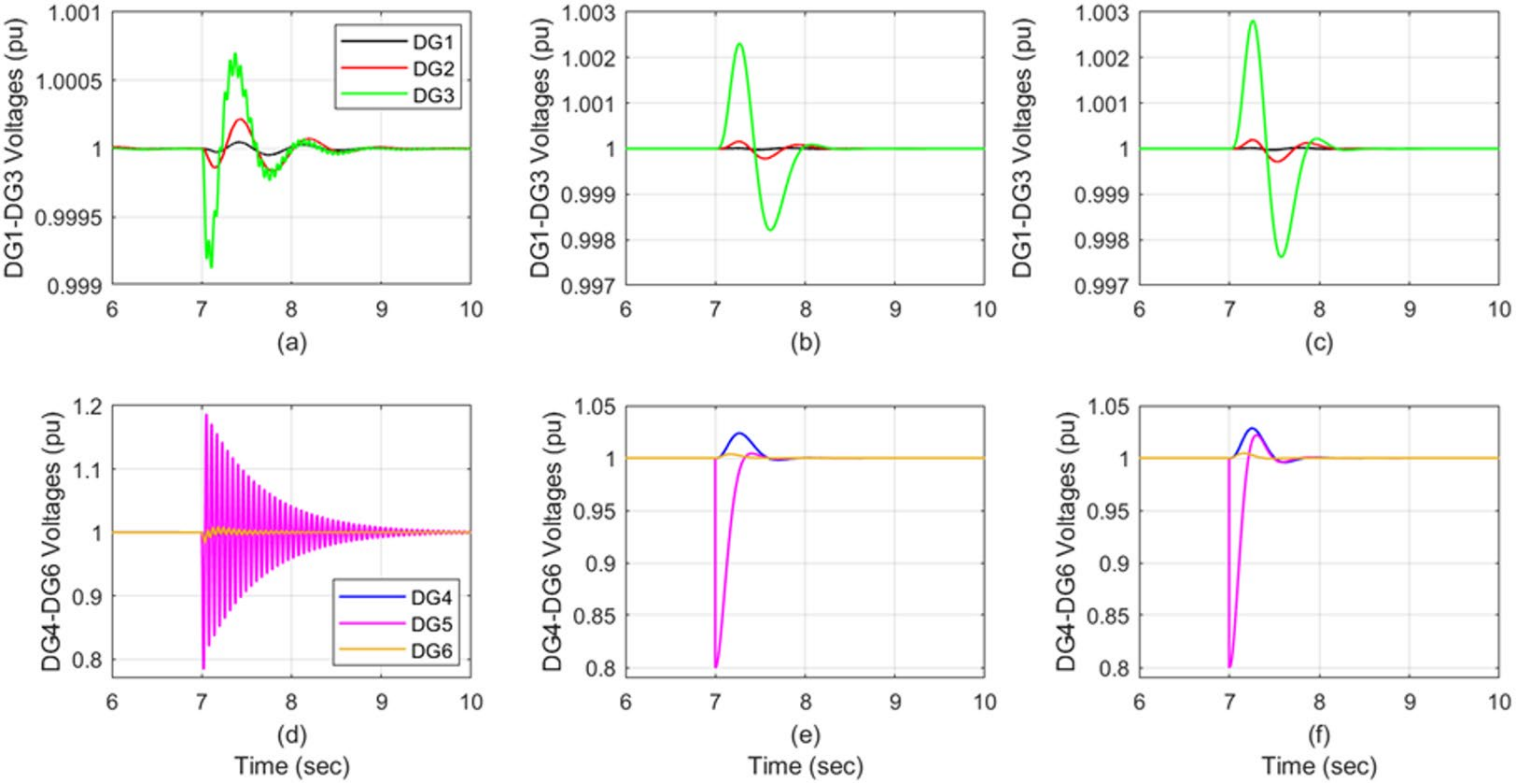
The DGs’ voltage response when DG_5_’s sensor effectiveness degrades from 100% to 80%. Auto-tune PI in (**a,d**), attractive ellipsoid in (**b,e**) and ADRC in (**c,f**).

A randomly selected sensor fault was applied to DG5 at time *t* = 7 s. It generated a random sensor fault involving a 20% degradation (80% effectiveness) in DG5. Figure 5 presents the DGs’ voltage response currently, comparing the auto-tuned PI (5a, 5d), the attractive ellipsoid (5b, 5e), and the ADRC (5c, 5f) control techniques. The auto-tuned PI method shows a significant impact from the fault, severely affecting both the faulty DG and the remaining five DGs. In sharp contrast, the ADRC and attractive ellipsoid techniques effectively isolate the fault, causing only slight, minimal effects on the other five DGs. Furthermore, the ADRC demonstrates a slightly faster response than the attractive ellipsoid method. Table 6 compares the total impact of each control technique on the faulty DG and the healthy DGs.

### Scenario 2: consecutive sensor degradations on two DGs

**Fig. 6:**
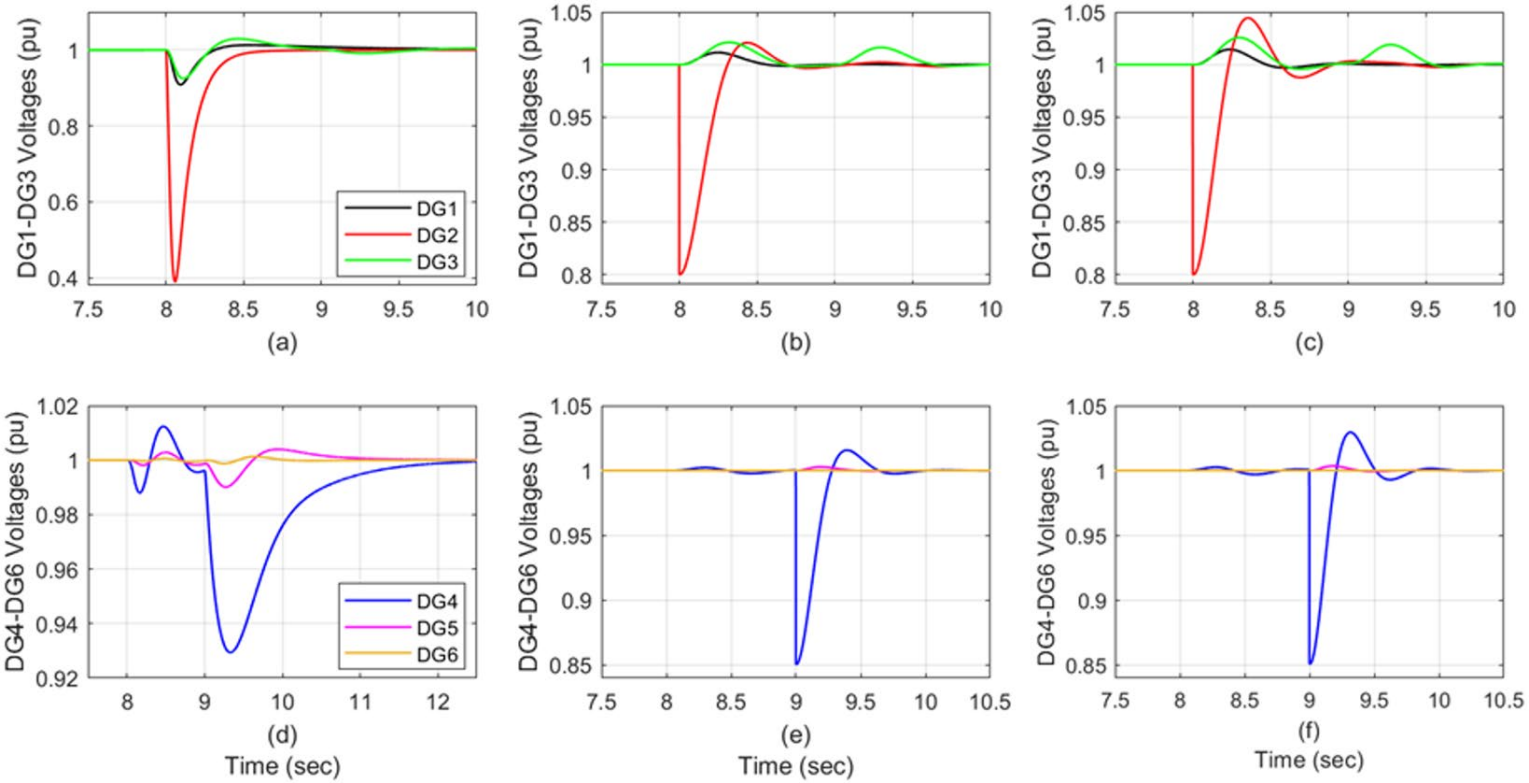
DG voltage under successive sensor degradation: DG2 (80%) followed by DG4 (85%); (**a,d**) auto-tune PI, (**b,e**) attractive ellipsoid, and (**c,f**) ADRC.

Randomly, two consecutive sensor failures were considered: DG2 at 80% effectiveness at time *t* = 8s and DG4 at 85% effectiveness at time *t* = 9s. Figure 5a and d illustrate the bus voltage behavior of the system using auto-tuned PI control, whereas Fig. 5b and e depict the voltage behavior using the attractive ellipsoid control method, and Fig. 5c and f illustrate the voltage behavior using the ADRC approach. The ADRC behaves as a disturbance rejection providing robust performance with limited impact for DG1 and DG3 despite the loss of sensor data. It is also obvious that the first fault in DG2 has a limited impact on DG1 and DG3 (Fig. 5c). The robust performance of the proposed controller remains consistent through the success of sensor failures on DG4 and its surrounding DGs. The effects on the faulty DGs and their neighboring units are detailed in Fig. 6, comparing the operation with the auto-tuned PI controllers (Fig. 6a, d), the attractive ellipsoid controllers (Fig. 6b, e), and the ADRCs (6c, 6f). Table 6 compares the total consequences of each control technique across the DGs, differentiating between the faulty and healthy units.

### **Scenario 3: simultaneous degradations on two DGs** (within the design control range in^15^ )

**Fig. 7:**
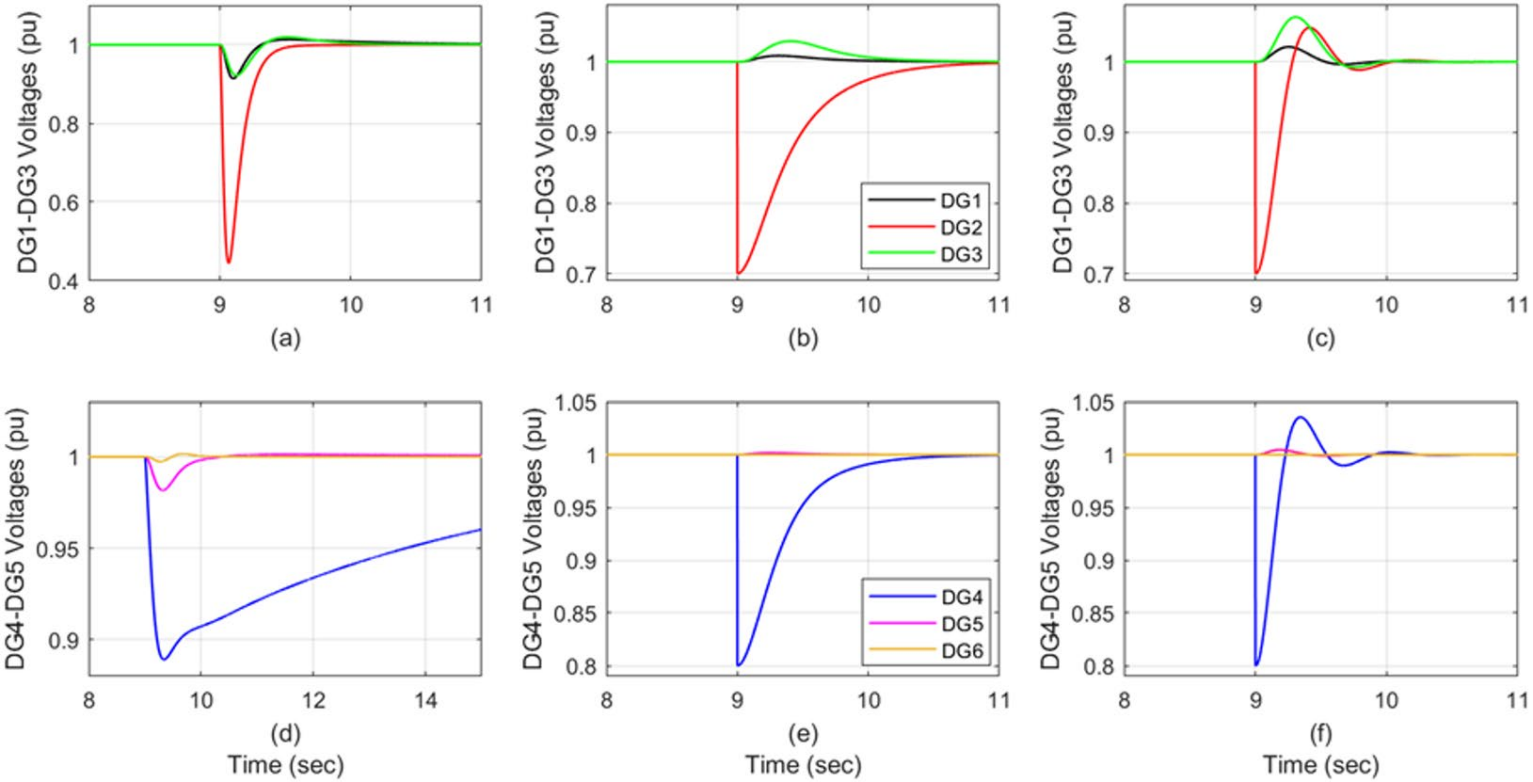
DG voltage under simultaneous sensor degradation: DG2 (70%) and by DG4 (80%); (**a,d**) auto-tune PI, (**b,e**) attractive ellipsoid, and (**c,f**) ADRC.

At time *t* = 9 s, sensors for DG2 and DG4 were selected randomly, demonstrating 70% and 80% effectiveness, respectively. When using the traditional auto-tuned PI control approach, both DG2 and DG4 experience simultaneous sensor failures. The attractive ellipsoidal control technique yields better results (Fig. 7b and e). However, the ADRC (Active Disturbance Rejection Control) technique is faster and provides better performance than both PI and attractive ellipsoidal control, despite resulting in two distinct, concurrent sensor faults in DG2 and DG4 (Fig. 7c and f). The total effect of each control technique on the system’s DGs, specifically differentiating between the faulty DG and the healthy DGs, is compared in Table 6.

### **Scenario 4: Simultaneous degradations on two DGs** (outside the design control range in^15^)

**Fig. 8:**
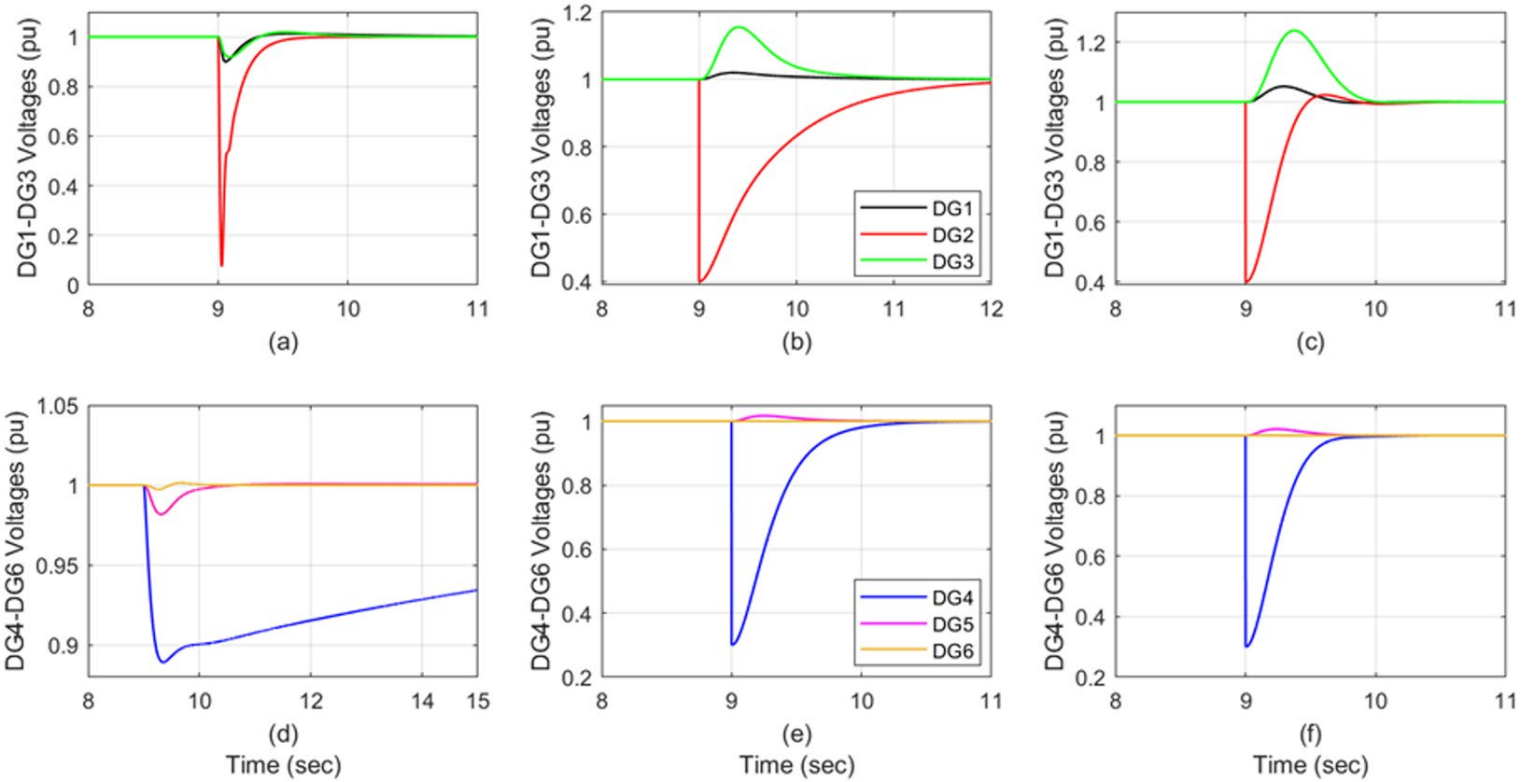
DG voltage under simultaneous sensor degradation: DG2 (40%) and by DG4 (30%); (**a,d**) auto-tune PI, (**b,e**) attractive ellipsoid, and (**c,f**) ADRC.

To ensure severe validation, the proposed system was tested under conditions where two DG sensors operated concurrently with effectiveness values significantly outside the specified control range. This involved the random selection of DG_2_ (40% effectiveness) and DG_4_ (30% effectiveness) simultaneously. The ADRC proposed technique successfully rejected the sensor disturbance and maintained perfect operation. As illustrated in Fig. 7c and f, the proposed trackers behaved with sufficient response time and had only a small fault impact on the neighboring DGs. In contrast, although the auto-tuned PI control was not able to deal with the disturbance (Fig. 8a and d), the ellipsoidal control shows a slower response compared to the ADRC (Fig. 8b and e). A comprehensive comparison between these three control techniques is depicted in Table 6. It should be noted that each simulation scenario was repeated three times to ensure repeatability. The reported values correspond to the average performance metrics across trials. The steady-state error values reported as “≈ 0.0” indicate numerical errors below 10⁻³ p.u., which are negligible within the simulation precision. The small variation observed across repeated tests confirms the consistency and reliability of the presented results.

**Table 6:** System dynamic characteristics during the four scenarios.

Case number	Output voltage	% of voltage sag during sensor degradation (%)			System dynamic characteristics								
					% Overshoot (%)			Settling time (s)			Steady state error (%)		
		PI-tuned	Ellipsoid	ADRC	PI-tuned	Ellipsoid	ADRC	PI-tuned	Ellipsoid	ADRC	PI-tuned	Ellipsoid	ADRC
Scenario 1 Case 1: sensor degradation in DG_1_ (75%)	DG_1_	41.7	25.015	25.012	≈ 0.0	≈ 0.0	1.132	2.542	0.521	0.367	0.014	≈ 0.0	≈ 0.0
	DG_2_	11.8	0.031	0.032	10.32	3.41	2.76	1.912	0.487	0.321	0.009	≈ 0.0	≈ 0.0
	DG_3_	1.34	0.019	0.021	4.78	0.728	0.627	1.321	0.395	0.287	≈ 0.0	≈ 0.0	≈ 0.0
	DG_4_	0.046	0.004	0.004	0.836	0.0324	0.0289	1.102	0.372	0.262	≈ 0.0	≈ 0.0	≈ 0.0
	DG_5_	0.023	0.0012	0.0013	0.215	0.00329	0.0345	1.078	0.303	0.215	≈ 0.0	≈ 0.0	≈ 0.0
	DG_6_	0.015	0.00031	0.0004	0.064	≈ 0.0	≈ 0.0	1.052	0.156	0.114	≈ 0.0	≈ 0.0	≈ 0.0
Scenario 1 Case 2: sensor degradation in DG_5_ (80%)	DG_1_	0.065	0.0073	0.0081	0.064	0.0064	0.012	1.052	0.341	0.272	0.0016	≈ 0.0	≈ 0.0
	DG_2_	0.115	0.015	0.0171	0.181	0.089	0.115	1.069	0.464	0.335	0.0015	≈ 0.0	≈ 0.0
	DG_3_	0.186	0.182	0.204	0.289	0.167	0.204	1.151	0.572	0.3981	0.0021	≈ 0.0	≈ 0.0
	DG_4_	2.624	0.324	0.351	1.52	1.99	2.01	1.632	0.478	0.382	0.067	≈ 0.0	≈ 0.0
	DG_5_	22.1	20.02	20.13	19.87	0.145	1.474	1.914	0.592	0.4215	0.066	≈ 0.0	≈ 0.0
	DG_6_	2.07	0.272	0.281	1.63	0.917	1.271	1.717	0.497	0.4017	0.0589	≈ 0.0	≈ 0.0
Scenario 2: Consecutive sensor degradations on two DGs	DG_1_	10.283	1.24	1.43	2.59	1.542	2.084	0.452	0.621	0.482	0.0671	≈ 0.0	≈ 0.0
	DG_2_	60.254	20.043	20.022	≈ 0.0	2.15	4.722	1.856	0.421	0.331	0.104	≈ 0.0	≈ 0.0
	DG_3_	9.961	0.91	1.02	3.97	2.063	3.272	0.631	0.765	0.5023	0.737	≈ 0.0	≈ 0.0
	DG_4_	7.798	15.003	15.017	1.167	2.086	3.591	2.921	0.3978	0.278	0.0943	≈ 0.0	≈ 0.0
	DG_5_	1.725	0.824	0.811	0.767	0.532	0.722	1.241	0.3482	0.274	0.0631	≈ 0.0	≈ 0.0
	DG_6_	0.965	0.672	0.542	0.541	0.367	0.4981	0.897	0.2963	0.2056	0.0135	≈ 0.0	≈ 0.0
Scenario 3: Simultaneous degradations on two DGs (within the design control range in^15^*)*	DG_1_	10.02	1.157	1.734	1.134	2.134	2.963	0.675	1.102	0.872	0.176	≈ 0.0	≈ 0.0
	DG_2_	56.93	30.09	30.008	≈ 0.0	≈ 0.0	6.472	0.925	1.642	0.421	1.205	≈ 0.0	≈ 0.0
	DG_3_	10.24	1.651	1.025	1.561	3.751	8.135	0.745	0.893	0.634	0.183	≈ 0.0	≈ 0.0
	DG_4_	12.58	20.12	20.07	≈ 0.0	≈ 0.0	3.769	12.89	1.493	0.359	1.198	≈ 0.0	≈ 0.0
	DG_5_	2.062	0.871	0.772	0.689	1.235	1.734	0.581	0.341	0.302	0.087	≈ 0.0	≈ 0.0
	DG_6_	0.974	0.475	0.341	0.451	1.178	1.265	0.499	0.214	0.109	0.042	≈ 0.0	≈ 0.0
Scenario 4: Simultaneous degradations on two DGs (outside the design control range in^15^*)*	DG_1_	9.96	1.25	1.12	1.023	5.203	7.062	0.723	1.205	0.694	0.934	≈ 0.0	≈ 0.0
	DG_2_	96.21	59.95	60.01	≈ 0.0	≈ 0.0	1.2636	0.872	2.754	0.547	1.992	≈ 0.0	≈ 0.0
	DG_3_	10.23	1.35	1.052	1.257	16.422	21.73	0.828	1.562	0.8027	1.024	≈ 0.0	≈ 0.0
	DG_4_	13.03	65.02	64.99	≈ 0.0	≈ 0.0	≈ 0.0	22.34	1.567	0.8346	2.191	≈ 0.0	≈ 0.0
	DG_5_	3.672	0.985	0.881	≈ 0.0	2.131	2.356	0.521	0.924	0.437	0.843	≈ 0.0	≈ 0.0
	DG_6_	3.192	0.873	0.657	≈ 0.0	1.785	1.983	0.243	0.651	0.1982	0.654	≈ 0.0	≈ 0.0

## Discussion

The case study results presented in Figs. 4, 5, 6, 7 and 8 and highlighted in Table 6 evidently illustrate the excellent performance of the proposed Active Disturbance Rejection Control (ADRC) method against both the auto-tuned PI and the Attractive Ellipsoidal controllers. The ADRC displayed great robustness, stability, and adaptability to all fault conditions while maintaining consistently a minor voltage deviation and almost zero steady-state error for all distributed generators connected to the microgrid. For each case study, the PI-tuned controller showed a very high dependence on accurate modeling of the system and extreme sensitivity to degradation in the sensor condition. As shown in the results, a minor reduction of only one sensor’s functionality resulted in a very significant drop in voltage, considerable settling time, and visible oscillation that spread to adjacent DG units. The Attractive Ellipsoidal controller was an improvement in this regard, as it showed much better damping characteristics and suppressed some of the fault propagation; however, its convergence was slower than the PI controller, and it exhibited a higher control effort requirement, especially during fast-changing fault conditions. On the other hand, the ADRC exhibited better fault tolerance through the extended state observer (ESO), which constantly estimated and compensated for unmeasured disturbances both accurately and in real time. This allowed for quick disturbance rejections and fault recovery, while maintaining the system voltage stability.

When DG1 had seen 25% sensor failure, the PI-tuned controller experienced a severe voltage sag of approximately 41.7% and took approximately 2.5 s to settle. The Attractive Ellipsoidal method, which limited the sag to about 25%, recovered in around 0.5 s, whereas the ADRC showed the best performance, with a minor overshoot and a settling time of about 0.37 s. A similar improvement can be seen in the event when the DG5 sensor was at 80% effectiveness, in which the PI controller showed significant oscillations and disturbances, while the ellipsoidal control limited the disturbance to one DG but was slow to respond. The ADRC recovered the voltage quickly, where the overshoot was below 1% with no steady-state error.

In response to successive sensor faults in DG2 and DG4, the behavior of the PI controller exhibited significant inter-DG interaction and an average settling time higher 1.8 s. The Ellipsoidal controller was able to recover the bus voltage with a longer settling time, whereas the ADRC was able to maintain all DG voltages within the accepted limits with an average settling time of less than 0.5 s, indicating its ability to deal with consecutive disturbances efficiently. For the fault scenario and under the design control (DG2 = 70%, DG4 = 80%), the ADRC again surpassed the other two controllers. While the PI approach produced voltage sags of more than 56% and oscillations that propagated to the neighboring DG units, the Ellipsoidal approach was able to limit the sag to approximately 30% but took a longer time to recover from the transient state. The ADRC was able to stabilize the microgrid quicker than both approaches, with minor overshoot and was able to get back to its steady state condition in a very short time.

The most challenging test was performed under conditions that included simultaneous faults occurring away from the design range, where DG2 and DG4’s sensor performance levels decreased to 40% and 30%, respectively. At these limits, the PI controller completely failed to regulate the system voltage, causing over a 90% sag. The Ellipsoidal controller showed substantial oscillations and long settling times. The ADRC, on the other hand, continued the stable operation, showing a quick recovery with a voltage sag of less than 60%. Even under such a challenging case, the ADRC was able to deal with the faults and did not allow faults to propagate neighboring DGs, demonstrating robustness and flexibility under extreme conditions.

The comparative data presented in Table 6 summarized the above findings. In each case, the ADRC consistently had the least overshoot, the shortest settling time, and no steady-state error. The PI controller demonstrated the least capability to tolerate fault perturbations; the autotune PI controller was poorly damped and very sensitive to the degradation of the sensor input. The Attractive Ellipsoidal controller was a significant improvement over the autotune PI controller, while being slower and less effective than the ADRC when challenged with complex or high complexity disturbances. The decentralized architecture of the ADRC and the minimal tuning requirements and real-time disturbance estimation show a distinct advantage during application in practice in DC microgrids. The ADRC eliminates the need for explicit fault detection or reconfiguration of the coordination model while ensuring stable voltage regulation and coordinated DG operation by making faults into compensable disturbances.

Although the primary validation focused on sensor degradation scenarios, it should be noted that ADRC inherently compensates for parameter perturbations and load transients through its lumped disturbance estimation mechanism. Variations in line resistance, converter parameters, or load steps are reflected in the disturbance term and are therefore rejected by the ESO in the same manner as sensor faults. Consequently, the disturbance-rejection capability demonstrated under sensor failure conditions extends to composite disturbances commonly encountered in practical microgrid operation.

## Conclusion

This study investigates a low-voltage DC microgrid equipped with a sensor fault-tolerant control scheme based on active disturbance rejection. The research outlines an overall system model and the essential analytical control equations needed to evaluate the performance of the proposed scheme under various sensor faults. The simulation results show that the proposed ADRC scheme provides significantly more fault-tolerant voltage regulation than the standard PI controller and attractive ellipsoidal controllers. The ADRC proved to keep the DG bus voltages within tolerable ranges between scenarios of single or consecutive and simultaneous sensor faults, leading to less voltage sag, overshoot, and settling times than the PI and attractive elliptic controllers. These major performance improvements are attributed to the extended state observer, which constantly estimates the combined lumped disturbances and sensor-fault effects on the closed-loop control. Conversely, the autotune PI controller was found to be very sensitive to parameter uncertainty and faults, and disturbances propagated among the units of the DC microgrid adversely, and the attractive ellipsoidal controller showed slower convergence and greater control effort. In general, this research shows that an ADRC can provide reliable, scalable, and computationally efficient decentralized fault-tolerant control of a DC microgrid, making it an excellent candidate for next-generation resilient DC distribution systems.

## Data Availability

The datasets used and/or analyzed during the current study available from the corresponding author on reasonable request.
